# Biology and Morphometric Characterization of the Invasive Pest *Spodoptera frugiperda* in Agricultural Regions of South Sulawesi, Indonesia: Implications for Pest Management

**DOI:** 10.1155/sci5/9175678

**Published:** 2025-12-22

**Authors:** Melina Melina, Sulaeha Sulaeha, Nurfauziyah Nurfauziyah

**Affiliations:** ^1^ Department of Plant Pest and Disease, Faculty of Agriculture, Hasanuddin University, Makassar, Indonesia, unhas.ac.id; ^2^ Postgraduate Student of Department of Plant Pest and Disease, Faculty of Agriculture, Hasanuddin University, Makassar, Indonesia, unhas.ac.id

**Keywords:** fall armyworm, life cycle, morphometric characterization, pest management, South Sulawesi

## Abstract

*Spodoptera frugiperda* (fall armyworm) is an exotic pest from the American continent that has invaded agricultural lands in Indonesia, particularly affecting maize crops. This study aimed to investigate the biological and morphometric characteristics of *S. frugiperda* larvae fed with baby corn under controlled laboratory conditions. Several biological parameters were observed, including the preoviposition, oviposition, and postoviposition periods, egg production and frequency, and the number of larval instars. The study also monitored the duration of the prepupal and pupal stages, mortality rates, sex ratios, adult lifespan, and body size. Results indicated that *S. frugiperda* exhibits high reproductive capacity, with females laying an average of 133.25 eggs that hatch within 1–2 days. Larvae underwent six instars over 14–16 days, and the pupal stage lasted 9–11 days before emerging as adults. Male adults lived for 6–8 days, while females survived longer, between 9 and 13 days. The total lifespan from egg to adult death averaged 30 days for males and 35 days for females. High mortality was observed in the pupal stage and the first larval instar. Morphometric data revealed gradual increases in larval length from the first instar (2.25 mm) to the sixth instar (32 mm), with pupae reaching 16 mm in length, and adults measuring 14 and 13 mm for males and females, respectively. The relatively small body size of *S. frugiperda* contributes to its ability to disperse and invade agricultural areas in Indonesia. This study provides valuable insights into the biology and morphometrics of *S. frugiperda*, which may serve as a foundation for developing sustainable pest management strategies in the region.

## 1. Introduction

Corn is a strategic commodity that contributes significantly to the economy and social identity of the Indonesian community. The growing demand for corn in the market necessitates the implementation of sustainable practices to increase production. To achieve this, the government prioritizes synergy between farmers and agricultural extension workers by expanding land in several regions of Indonesia to support the growth of corn production [1–3]. However, attacks by plant pests are an obstacle that has an impact on corn production. *Spodoptera frugiperda* is currently a pest species with a high population and dominates attacks on several corn plantations in Indonesia [4, 5]. This insect pest attacks more than 80 plant species, mainly causing damage to cereal crops, such as corn. *S. frugiperda* is an insect native to South and Central America that regularly migrates to Canada and has been recorded moving south during the winter [6]. In January 2016, this pest appeared in Southwest Nigeria and Ghana, then spread rapidly until August 2017, and was confirmed in 28 African countries [7]. In 2018, *S. frugiperda* was reported to have entered the Asian region, precisely in India, and continued to spread to Southeast Asia, including Thailand and Myanmar, in 2019 [8, 9]. Based on the morphological characteristics, the pest was in Lampung Province, Indonesia, in May 2019 [10]. In general, the distribution of *S. frugiperda* is largely influenced by the host plants. The pest reproduces rapidly and can fly up to 100 km per night, facilitating easy spread to other areas. Tropical climate conditions in corn‐producing areas, such as the Asian continent, are suitable for supporting the emergence and reproduction of this pest [11–13].

*S. frugiperda* has the potential to cause corn yield losses of 8.3–20.6 million tons per year in 12 corn‐producing African countries. This figure is equivalent to 21%–53% of annual production, with losses reaching US $2.48–6.19 billion when there is no rapid control [7]. In Udaipur, India, about 75%–80% of the damage was confirmed at the knee‐high growth stage, which is the most vulnerable and critical phase for control [12]. In Indonesia, the percentage of *S. frugiperda* attacks is quite high, reaching 72%–98% in Lombok [14] and 46.44%–97.33% in Takalar, South Sulawesi, at plant age 3–4 weeks [15]. The vast area of corn farming land in Indonesia makes this region vulnerable to *S. frugiperda* attacks. This pest has the potential to significantly reduce corn productivity and attack other higher plants, thereby necessitating serious attention. Due to the high migration and fecundity capabilities, voracious larval feeding patterns, and wide host range, *S. frugiperda* is a global threat that has a significant impact on economic losses in the agricultural sector [16]. Therefore, this research aims to determine the biological and morphometric aspects of *S. frugiperda* reared on baby corn feed under controlled laboratory conditions. Baby corn was chosen due to its suitability as a natural host for *S. frugiperda* and its balanced nutritional content, which supports optimal growth and development. Previous studies reported that baby corn contains relatively high protein (17.96/100 g) with a digestibility of 72.18%, as well as significant amounts of carbohydrates, essential minerals, and vitamins [17]. It is hypothesized that *S. frugiperda* reared on baby corn will exhibit a short life cycle and high reproductive potential, with distinct biological and morphometric patterns across developmental stages. The results are expected to provide valuable insights into the pest’s growth, reproductive rates, and life cycle, which are crucial for designing targeted pest control strategies in South Sulawesi. By understanding these biological traits, this study aims to inform more effective and sustainable pest management practices, focusing on critical life stages to improve crop protection, particularly for maize, and to serve as a basis for future research on *S. frugiperda* control.

## 2. Materials and Methods

### 2.1. Insect Rearing

The *S. frugiperda* larvae used in this research were obtained from corn plantations in Takalar Regency, South Sulawesi Province, Indonesia, which is known as the largest maize‐producing region in South Sulawesi. The larvae were kept separately in plastic cup containers under controlled temperature and relative humidity conditions of ±27°C and ±54% RH, respectively, in the laboratory of the Department of Plant Pests and Diseases, Faculty of Agriculture, Universitas Hasanuddin. *S. frugiperda* larvae were individually fed baby corn, and once the pupae were formed, the pests were transferred into plastic containers filled with moistened sterile sand. The adult insects were then placed into a 40 × 40 × 40 cm cage containing two pairs of adults. The cage was lined with cotton soaked in 10% honey solution as a nutrient source, and cotton was also attached to the top of the cage. Additionally, pieces of corn leaves were inserted into the cage for egg‐laying, which were later used for biological observations of *S. frugiperda* insects.

#### 2.1.1. Preoviposition Period, Oviposition Period, Postoviposition Period, Number of Eggs, and Frequency of Oviposition

A total of two pairs of *S. frugiperda* adults (F1) were placed in the cage, where copulation occurred. Observations began after copulation, focusing on the preoviposition, oviposition, and postoviposition periods, as well as the number of eggs laid and the frequency of oviposition. This experiment was conducted with four replications, each consisting of eight pairs of adults.

#### 2.1.2. Egg Stage, Stage of Each Larval Instar, Number of Larval Instar Stages, Prepupal Stage, Pupal Stage, and Percentage of Mortality for Each Stage

Observations of developmental stages were carried out daily, and the laid eggs were transferred to containers containing baby corn. Groups of eggs of the same age were observed by counting hatched and unhatched eggs. The larval instars were determined based on molting (ecdysis), with the first instar lasting from egg hatching until the first molt. Mortality was calculated from the number of dead larvae. The subsequent instars were recorded in the same way, by noting the intervals between molts and the number of dead larvae. The prepupal stage was defined as the period from the onset of puparium formation, whereas the pupal stage was defined as the duration between pupation and adult emergence.

#### 2.1.3. Sex Ratio and Adult Lifespan

The observations used adults derived from the experiment at each larval instar stage. The comparison between males and females was carried out only on insects that successfully developed into adults. Male and female adults were distinguished by the arrangement of wing colors. The lifespan of the adults was determined by recording the duration from adult emergence from the pupal stage until death.

### 2.2. Data Analysis

The data obtained from daily biological observations were recorded and entered into the MS Excel 2010 software program. These collected data were then processed by calculating the average from four repetitions for each biological parameter that has been carried out.

## 3. Results

**Figure 1:**
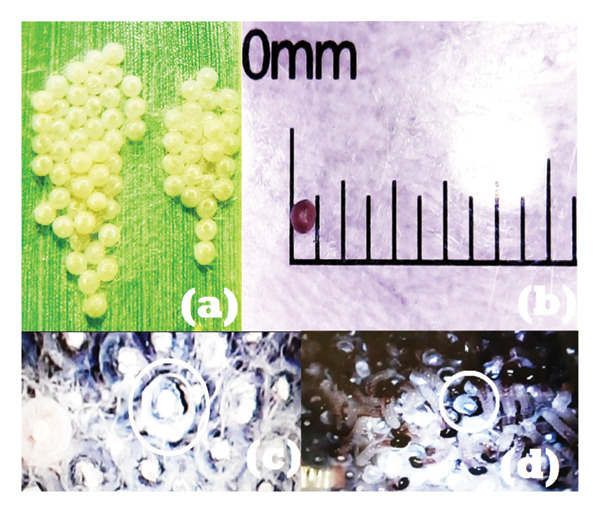
*S. frugiperda* egg group (a), egg size (b), eggs about to hatch (c), and hatched eggs (d).

The eggs of *S. frugiperda* were laid in groups, as shown in Figure 1(a). Furthermore, the number of egg groups laid in one female individual ranges from 1 to 3 groups, with an average number of 133.25 per female individual, as shown in Table 1. The eggs are round, yellow–brown in color, with an average size of 0.475 mm. Before hatching, the color of the eggs turns blackish as a sign of mature embryos. Eggs hatch in 1–2 days at an average temperature of 27.5°C and humidity of 54%, as shown in Figures 1(c) and 1(d). The result of this research showed that hatching time was influenced by temperature and humidity. Low temperatures slow down development and also increase egg mortality. According to Du Plessis et al., a temperature of 18°C produced a hatching time of 6–7 days, while 26°C–32°C was within 2–3 days [18].

**Table 1:** Mortality rates at egg, larval instar, prepupal, and pupal stages of *S. frugiperda* per female adult.

Stadium	Number of individuals/female adult	Number of dead individuals/female adult	±SD	Mortality (%)
Egg	133.25	20	13.10	15.01
Larvae 1	113.25	37.5	32.78	33.11
Larvae 2	75.75	9.5	8.69	12.54
Larvae 3	66.25	4125	2.43	6.23
Larvae 4	62.25	3125	2.17	5.02
Larvae 5	59.00	1625	0.85	2.75
Larvae 6	57.50	0.5	1.00	0.87
Prepupae	57.00	4875	1.25	8.55
Pupae	52.00	19,125	13.81	36.78

High mortality at each stage resulted in only a small proportion of *S. frugiperda* eggs reaching the adult stage, as shown in Table 1. The high mortality of pupae and first instar larvae was due to inadequate supportive environmental conditions. Intraspecific cannibalism often occurs among young generation larvae [19] and is often observed even in the presence of sufficient food [20–22]. Cannibalism mainly reduces density in the young generation and begins to decrease after the third instar [23]. During cannibalism, aggressive and defensive actions drain physical energy, worsen nutrient deficiencies, and increase the risk of injury and death [24]. The main causes of high mortality in this stage include environmental factors, nutrient availability, and physiological weaknesses of larvae.

**Figure 2:**
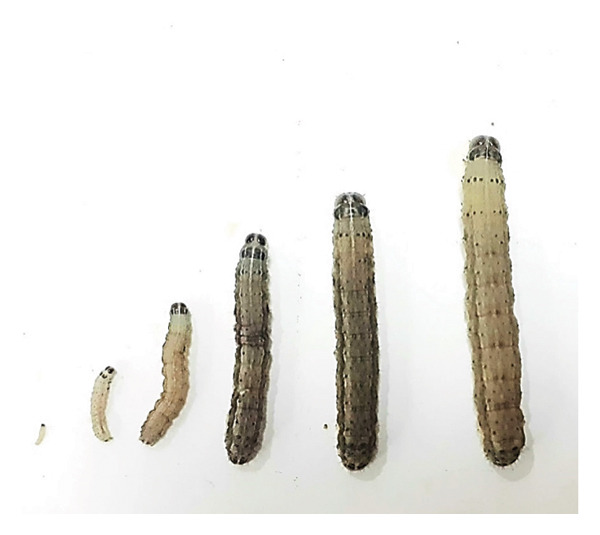
*S. frugiperda* larvae Instar 1–Instar 6.

*S*. *frugiperda* larvae possessed three pairs of true legs and four pairs of prolegs. The larvae underwent six instars (Figure 2) before entering the prepupal stage, which was characterized by five molting events, as indicated by the presence of exuviae in the rearing container. Table 2 presents the duration of the larval stage from the first to the sixth instar, ranging from 14 to 16 days with an average of 14.5 days. The duration of the larval stage was influenced by temperature and humidity. According to research by Assefa and Ayalew in 2019 [25], the larval stage lasts 14 to 30 days during the summer and winter, respectively. In the last instar larvae, the time required is longer than that in the other instars. This is related to a decrease in the relative consumption rate, showing a decline in the digestive ability of the larvae as the pupal stage advances. *S. frugiperda* larvae that have reached the final instar will experience weight loss before entering the pupal stage, which is known as the critical weight stage [26].

**Table 2:** Duration of egg stage, larval instars, prepupa, and pupa of *S. frugiperda.*

Stadium	Range (days)	Average (days)	±SD
Egg	1–2	1.87	±0.16
Larvae 1	3–4	3.5	±0.58
Larvae 2	2–3	2.5	±0.58
Larvae 3	3–4	3.5	±0.58
Larvae 4	2–4	3.3	±0.96
Larvae 5	3–4	3.3	±0.5
Larvae 6	3–4	3.8	±0.5
Prepupae	1–2	1.45	±0.29
Pupae	9–11	10	±0.96

**Figure 3:**
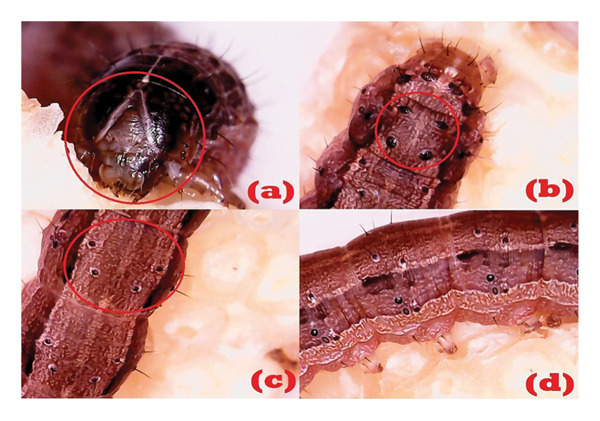
“Y” pattern on the caput (a), black spots appear on the abdomen of the 8^th^ segment in a square shape (b), black spots in a trapezoid shape on the abdomen of segments 1–7 and 9 (c), and lines on the lateral part of the abdomen (d).

From the morphological observation of *S. frugiperda* larvae, the first instar larvae were observed to have a brownish‐white body and a black head. In the second instar, the larvae are larger with a black spot pattern that can be seen using a microscope. In the third instar, the brown body with an inverted “Y” pattern on the head begins to be visible. Instars four, five, and six show a darker brown body color with more visible lateral lines and spots in Figure 3.

**Figure 4:**
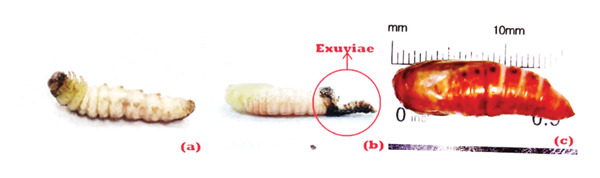
Prepupae (a), newly formed pupae (b), and *S. frugiperda* pupae (c).

*S. frugiperda* larvae are less active before transforming into pupae, showing the transition to the prepupal stage. Without soil, the larvae wrap their bodies using leftover feces or corn feed to form a pupal house. In field conditions, pupae are formed by binding soil particles or leaves with silk [25]. In the prepupal stage, the body of the larva changes color from brown to whitish and shrinks, as shown in Figure 4(a). This process typically lasts 1–2 days, with an average duration of 1.45 days, resulting in pupae that exhibit a greenish‐white coloration, which subsequently transitions to reddish brown, as shown in Figures 4(b) and 4(c). Major changes occur during the pupal stage to the adult, including a transformation of the mouthparts from biting–chewing in larvae to sucking in adults. This complex process causes a high mortality of 36.78%, as shown in Table 1.

A significant number of pupae did not successfully develop into adults, due to less‐than‐supportive environmental conditions. Pupal development into the adult lasts 9–11 days, averaging 10 days, as presented in Table 2. Differences in maintenance temperature can affect the biological activity of *S. frugiperda.* A temperature of 27°C is the most suitable temperature for *S. frugiperda* in carrying out the life cycle [27]. Furthermore, the pupae period of *S. frugiperda* varies based on temperature, ranging from 28 to 34 days at 18°C to 7–9 days at 32°C [18]. In addition to temperature and humidity, the type of larval feed also affects the development of pupae. The pupae period varies depending on the food source, lasting 8.1 and 10.18 days when fed corn [28] and soybean leaves, respectively [29].

The morphometric measurements obtained in this study, as detailed in Table 3, are generally consistent with the report of Deole and Paul [30], but slightly different from the explanation of Assefa and Ayalew [25], with larger results. The size of larvae and pupae varies due to differences in regions. Therefore, environmental conditions affect the morphometrics of larvae. Differences in geographical location may have contributed to the similarities in morphometric results between the research by Assefa and Ayalew [25] in Ethiopia, as well as Deole and Paul [30]. In addition, nutritional factors are important for the growth and development of *S. frugiperda*, and the nutritional conditions consumed by insects determine the body size, development, and reproductive processes [31]. Pupae whose larvae were fed corn leaves averaged 16.21 mm in size, significantly larger than those fed soybean leaves, which averaged 14.79 mm [26, 32].

**Table 3:** Morphometric measurements and descriptions of eggs, larval instars, and pupae of *S. frugiperda.*

Stadium	Length size (mm)	±SD	Description
Egg	0.475	±0.05	Round with an initial light yellow color which then changes color to brownish
Larvae 1	2.25	±0.26	Body color is brownish white, with a black head
Larvae 2	4.6	±0.42	The body color is brownish white with a dot pattern on the body that begins to appear
Larvae 3	12.25	±1.26	A brownish body color and a “Y” pattern on the head begin to appear
Larvae 4	20.75	±1.50	Brown body color and there is a “Y” pattern on the head, and on the abdomen of Segment 8, there are dots with a visible square pattern
Larvae 5	26	±0.82	Brown body color and there is a “Y” pattern on the head, and on the abdomen of Segment 8, there are dots with a visible square pattern with a larval length that is longer than the previous instar
Larvae 6	32	±1.83	Brown body color and there is a “Y” pattern on the head, and on the abdomen of Segment 8, there are dots with a visible square pattern with a larval length that is longer than the previous instar
Pupae	16	±0.82	The pupae is oval with a dark red color

**Figure 5:**
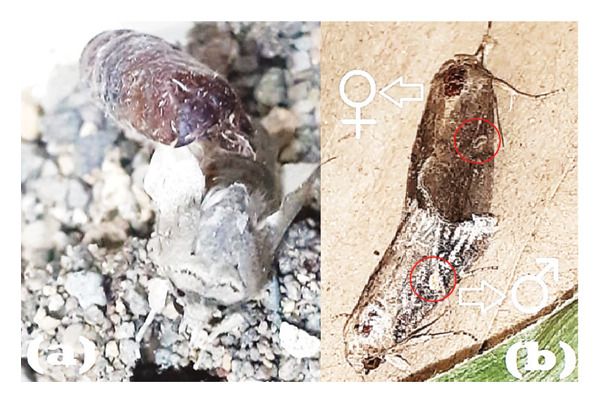
Newly emerged adult from the pupa (a); male adult and female adult (b).

The transition from pupae to adults was generally observed in the morning and evening. Newly emerged adults had two pairs of folded wings (Figure 5(a)). Furthermore, the wings of female adults were darker than those of males, as shown in Figure 5(b). Male wings were brown with a round yellowish‐orange pattern and white lines at the tips, whereas female wings were dark brown with faint white patterns. These characteristics were consistent with the literature on *S. frugiperda* adults, which reports that male forewings are typically brown and gray, decorated with triangular white spots near the middle and at the wing tips. Meanwhile, the wings of the female were dull brown with faint markings. In both sexes, the hindwings had a short dark border and a variable silvery white coloration [12, 32].

A comparison of male and female adults of *S. frugiperda* revealed that the number of female adults was higher, with a male‐to‐female sex ratio of 1:1.46, as shown in Table 4. The body size of male and female adults was nearly identical, with a difference in body length of only 1 mm, where the male adult was slightly longer, as shown in Table 5. Both forewings and hindwings measured 15 and 11 mm in length, respectively. However, the hindwings were broader than the forewings, exhibiting pronounced sexual dimorphism, with male adults having hindwings that were 2 mm wider than those of females. The hindwings are folded and covered by the forewings when at rest.

**Table 4:** Sex ratio of *S. frugiperda* per female individual.

Gender	Number of individuals
Male	13.38
Female	19.5

**Table 5:** Morphometrics of male and female adults of *S. frugiperda.*

Adult	Body parts	Size (mm)
Male	Body length	14
	Front wing length	15
	Rear wing length	11
	Front wing width	5
	Rear wing width	8

Female	Body length	13
	Front wing length	15
	Rear wing length	11
	Front wing width	5
	Rear wing width	6

The observations of the preoviposition, oviposition, and postoviposition periods of *S. frugiperda* are shown in Table 6. The female adults exhibited a longer oviposition period. A single female adult laid 1–3 egg batches, with an average of 133.25 eggs produced per female. Xia et al. [33] reported that the fecundity and age of *S. frugiperda* moths are inversely proportional to temperature. The optimal temperature for oviposition was 22°C, where females can lay an average of 156.6 eggs per day, explaining the nocturnal egg‐laying activity in tropical climates [33]. Based on the observation results, *S. frugiperda* adults were reported to actively copulate and lay eggs at night and in the morning. Females usually lay eggs on corn leaves but have also been found on other surfaces, such as duct tape, gauze, and rice paper. According to Silva et al. [34], female *S. frugiperda* laid eggs on all available hosts. For example, after soybean or cotton harvest, the growing grass also becomes a substrate for egg laying, thereby supporting the long‐term survival of the pest and increasing flexible host variation [34]. Host‐hunting behavior of insects was influenced by infochemical compounds in interactions with plants [35]. Research by Nurkomar et al. [36] showed that *S. frugiperda* was a potential alternative host because the pest could survive on 14 species of host plants commonly cultivated in Indonesia. The presence of these hosts contributed to the occurrence of attacks in corn fields. Similar research by Sharma et al. [37] confirmed the high potential for population growth of *S. frugiperda* on plants other than corn, showing the adaptation in maintaining the population.

**Table 6:** Duration of preoviposition, oviposition, and postoviposition periods of *S. frugiperda.*

Stadium	Range (days)	Average (days)	±SD
Preoviposition	3–6	4.25	±1.41
Oviposition	4–7	6	±1.50
Postoviposition	3–6	4.75	±1.26

Observation results showed that female and male adults live 9–13 and 6–8 days, with an average of 11 and 7 days, respectively. The total lifespan of insects from eggs to adults’ death is listed in Table 7. The life cycle of *S. frugiperda* was quite short with a high fecundity rate, enabling easy domination of agricultural cultivation areas. Climate conditions significantly affect the biological activity of *S. frugiperda*, showing that a lower temperature implies a longer life cycle [18, 27]. *S. frugiperda* cannot survive in harsh conditions, such as low winter temperatures, due to diapause, thereby triggering migration patterns [38]. The population of an insect varies in each type of region, influenced by factors, such as location, altitude, temperature, humidity, and the type of host plant available [39]. Indonesia, a tropical country with relatively warmer temperature conditions, supports the development of *S. frugiperda* populations. However, the stable average air temperature throughout the year, with increased rainfall, causes a highly favorable environment for the survival of *S. frugiperda* [40]. The findings of this study have significant implications for pest management strategies in South Sulawesi, Indonesia, and other regions of Southeast Asia. The short life cycle, high reproductive rate, and adaptability of *S. frugiperda* observed under local conditions suggest that control efforts should focus on early detection and management targeting the first larval instar, which experiences the highest mortality. Understanding the distinctive morphological and biological characteristics of the *S. frugiperda* population in South Sulawesi provides an important foundation for designing region‐specific integrated pest management (IPM) programs, including determining the appropriate timing for releasing natural enemies, using resistant maize varieties, and applying cultivation practices that can disrupt the pest’s reproductive cycle. These insights can support the development of more sustainable and environmentally friendly pest management strategies for *S. frugiperda* in Indonesia and other regions of Southeast Asia.

**Table 7:** Total lifespan of male and female *S. frugiperda* insects.

Gender	Range (days)	Average (days)	±SD
Male	27–33	30	±3.32
Female	30–38	35	±2.65

## 4. Conclusion

In conclusion, *S*. *frugiperda* exhibits high reproductive capacity and a short life cycle. Female adults produced an average of 133.25 eggs, which hatched within 1–2 days. Larvae passed through six instars over 14–16 days, followed by a pupal stage lasting 9–11 days before emerging as adults. Male adults lived for 6–8 days, whereas females survived longer, between 9 and 13 days. The total lifespan from egg to death averaged 30 days for males and 35 days for females. Notably, high mortality occurred during the pupal stage and the first larval instar. Larvae exhibited progressive growth from 2.25 mm in the first instar to 32 mm in the sixth instar. Pupae measured approximately 16 mm in length, while adult males and females measured 14 and 13 mm, respectively. The relatively small body size of *S. frugiperda* facilitates its dispersal and invasion of agricultural areas in Indonesia. Overall, insights into the biological and morphometric characteristics of *S. frugiperda* provide an essential foundation for developing sustainable pest management strategies.

## Conflicts of Interest

The authors declare no conflicts of interest.

## Funding

No funding was received for this manuscript.

## Data Availability

The data that support the findings of this study are openly available in Research Gate at https://www.researchgate.net/profile/Melina-Melina-5.
